# Extraction of lumenless pacing leads from the His bundle and left bundle branch area: outcomes of the high-volume centre

**DOI:** 10.1093/europace/euae213

**Published:** 2024-08-13

**Authors:** Rafal Gardas, Danuta Loboda, Jolanta Biernat, Tomasz Soral, Piotr Kulesza, Sylwia Gladysz-Wanha, Michal Joniec, Mateusz Sajdok, Kamil Zub, Krzysztof S Golba

**Affiliations:** Department of Electrocardiology and Heart Failure, Medical University of Silesia, Katowice, Poland; Department of Electrocardiology, Leszek Giec Upper-Silesian Medical Centre of the Silesian Medical University in Katowice, Ziolowa 45/47, Katowice 40–635, Poland; Department of Electrocardiology and Heart Failure, Medical University of Silesia, Katowice, Poland; Department of Electrocardiology, Leszek Giec Upper-Silesian Medical Centre of the Silesian Medical University in Katowice, Ziolowa 45/47, Katowice 40–635, Poland; Department of Electrocardiology and Heart Failure, Medical University of Silesia, Katowice, Poland; Department of Electrocardiology and Heart Failure, Medical University of Silesia, Katowice, Poland; Department of Electrocardiology and Heart Failure, Medical University of Silesia, Katowice, Poland; Department of Electrocardiology and Heart Failure, Medical University of Silesia, Katowice, Poland; Department of Electrocardiology and Heart Failure, Medical University of Silesia, Katowice, Poland; Department of Electrocardiology and Heart Failure, Medical University of Silesia, Katowice, Poland; Department of Electrocardiology and Heart Failure, Medical University of Silesia, Katowice, Poland; Department of Electrocardiology and Heart Failure, Medical University of Silesia, Katowice, Poland; Department of Electrocardiology, Leszek Giec Upper-Silesian Medical Centre of the Silesian Medical University in Katowice, Ziolowa 45/47, Katowice 40–635, Poland

**Keywords:** Conduction system pacing, His bundle pacing, Lead extraction, Left bundle branch area pacing

## Abstract

**Aims:**

The application of conduction system pacing (CSP) in clinical practice is growing, and the need for lead extraction will also increase. The data on outcomes and safety of CSP lead extraction are limited. The aim of this study was to assess procedural outcomes and safety of CSP lead removal.

**Methods and results:**

Forty-seven patients from the EXTRACT Registry with the indication for CSP lead removal were enrolled in the study conducted at the Department of Electrocardiology in Katowice, Poland. Extraction technique, outcomes, safety, and complication were evaluated. Forty-three (91.5%) leads were successfully removed, and 41 (87.2%) were removed with traction only. The dwelling time of 28 extracted leads was longer than 1 year, and the oldest extracted lead was implanted for 89 months. Seven (14.9%) leads were removed from the left bundle branch (LBB) area and 36 from the His bundle (HB). Transient complete atrioventricular block occurred during the procedure in two patients. In 27 out of 31 attempts (87.1%), new CSP leads were implanted: nine (33.3%) HB pacing leads and 18 (66.7%) LBB area pacing leads.

**Conclusion:**

The CSP lead extraction is safe and feasible with a low complication rate and high rate of CSP lead reimplantation.

**What’s new?**

- Transvenous lead extraction of lumenless leads from the His Bundle is feasible and safe.
- Simple traction is effective in the majority of cases, but complete removal failed in ∼10% due to subclavian entrapment. The use of passive sheath as a primary approach should be considered, especially in patients with severely compromised subclavian vein patency.
- Conduction system pacing lead reimplantation is feasible.

## Introduction

Pacemaker lead complications affect about 4–5% of implanted leads^1,2^ and, apart from infection, are one of the main reasons for revision procedures.^3^ Conduction system pacing (CSP), with His bundle pacing (HBP) or left bundle branch area pacing (LBBAP), is becoming a widely used alternative for right and left ventricular pacing.^4^ However, the mid-term and long-term performance of HBP leads demonstrates higher complication rates,^5^ mainly due to high and unstable pacing thresholds.^6^ Compared with HBP, LBBAP leads exhibit better performance,^7,8^ but a rapid rise in LBBAP lead implantations may be accompanied by an increasing number of leads requiring extraction.

This study presents the results of CSP lead extraction from a high-volume centre.

## Material and methods

We present an analysis of CSP lead removal procedures included in the EXTRACT Registry (ClinicalTrials.gov ID NCT05775783) and the prospective registry of conduction system pacing KatHisREG (KNW/0022/KB/17/18). Lead removal procedures were taken into analysis at least 1 month after the CSP lead implant. Depending on the dwell time of the CSP lead, the studied population was divided into two subgroups: (1) up to 1 year and (2) more than 1 year. The definitions of indications, procedures, and success rate assessment were adopted from the Heart Rhythm Society consensus statement on cardiovascular implantable electronic device lead management and extraction^9^ and the European Heart Rhythm Association consensus on designing scientific studies, reports, and registries relating to lead extraction, which was announced a year later.^10^ We performed 1652 implantations of CSP systems, including 723 LBBAP (43.8%), from December 2015 to February 2024, with periprocedural success in 1312 (79.4%) patients. Forty-seven required CSP lead explant or CSP lead extraction (seven with LBBAP) in 13 (5–40) months after implantation.

Indications for lead removal included pocket infections, lead-related endocarditis, and lead dysfunction such as sensing issues, high capture thresholds, or lead fracture.

Definitions of procedures:

- Lead explant: A lead removal procedure using simple traction techniques and all removed leads have been implanted for less than 1 year.
- Lead extraction: Lead removal procedure with removal of at least one lead implanted for more than 1 year, or a lead, regardless of implant duration, requiring the assistance of specialized equipment.
- Complete procedural success: Removal of all targeted leads without permanently disabling complication or procedure-related death.
- Clinical procedural success: Retention of a tip or a small portion of the lead (<4 cm) that does not negatively impact the outcome goals of the procedure and with no permanently disabling complication or procedure-related death.
- Procedure failure: Inability to achieve either complete procedural or clinical success or the development of any permanently disabling complication or procedural-related death.

Each procedure was initiated using the single traction method. After the lead was dissected free from the tissue in the pocket, gentle traction was applied with concomitant counterclockwise lead rotation. If this attempt was insufficient, mechanical polypropylene extraction sheaths (Byrd, Cook Medical, Leechburg, PA, USA) and lead extender (Bulldog, Cook Medical, Leechburg, PA, USA) were used. We recorded all the complications that occurred during the procedure.

### Statistical analysis

Continuous variables were expressed as means ± SD or median (IQR). The Shapiro–Wilk test was used to determine a normal distribution for continuous variables. The Mann–Whitney signed-rank test was used to compare data between groups for nonparametric data. Categorical data were presented as numbers and percentages and compared using the *χ*^2^ test.

Analyses were performed using MedCalc Statistical Software version 20.112 (MedCalc Software Ltd, Ostend, Belgium; https://www.medcalc.org; 2022).

## Results

### Baseline characteristics

Between April 2018 and March 2024, 47 lead removal procedures were performed. Twenty-eight (59.6%) were done after 1 year from lead implantation. The median age of the patients was 71.00 (62.00–76.00) years, and 33 (70%) were men. Except for sex and left ventricle ejection fraction, baseline characteristics were not different between the two groups (*Table 1*). The most common indication for pacing was atrioventricular block. High pacing threshold was the predominant reason for lead removal in 39 (83.0%) patients, followed by infection in 4 (8.5%: three local pocket infections and one endocarditis) patients, micro-dislodgement in two (4.3%) patients, atrial oversensing not manageable with reprogramming in one (2.1%), and lead fracture in one (2.1%).

**Table 1:** General characteristics

	All (*n* = 47)	Group 1 (lead dwell time 6–12 months) (*n* = 19)	Group 2 (lead dwell time over 12 months) (*n* = 28)	p
Age (years)	71.00 (62.00–76.00)	72.00 (62.00–78.00)	70.50 (62.50–75.00)	0.4
Sex (male)	33 (70.2)	10 (52.6)	23 (82.1)	0.03
Indication for pacing SND AVB Resynchronization	8 (17.0) 22 (46.8) 17 (36.2)	4 (21.1) 10 (52.6) 5 (26.3)	4 (14.2) 12 (42.9) 12 (42.9)	0.2
Indication for removal Infection High threshold Sensing issues Lead dislodgment Lead fracture	4 (8.5) 39 (83.0) 1 (2.1) 2 (4.3) 1 (2.1)	2 (10.5) 14 (73.7) 1 (5.3) 2 (10.5) 0	2 (7.1) 25 (89.3) 0 0 1 (3.6)	0.2
Threshold At implant At removal	1.50 (1.00–1.875) 3.50 (2.313–4.50)	1.50 (1.00–1.737) 3.00 (1.825–4.625)	1.60 (1.00–1.95) 3.75 (2.75–4.50)	0.5 0.3
Devices PM-VVI PM-DDD ICD-DDD CRT-P CRT-D	8 (17.0) 20 (42.6) 3 (6.4) 5 (10.6) 11 (23.4)	2 (10.5) 10 (52.6) 2 (10.5) 1 (5.2) 4 (21.1)	6 (21.4) 10 (35.7) 1 (3.6) 4 (14.3) 7 (25.0)	0.5
LVEF	49.00 (33.00–60.25)	56.00 (48.25–64.25)	45.00 (29.50–55.50)	0.02
CHF	22 (46.8)	6 (31.6)	16 (57.1)	0.09
CCD	24 (51.1)	9 (47.4)	15 (53.6)	0.7
Arterial hypertension	30 (63.8)	11 (57.9)	19 (67.8)	0.5
Diabetes mellitus	23 (48.9)	8 (41.1)	15 (53.6)	0.4
Chronic kidney disease	13 (27.7)	3 (15.8)	10 (35.7)	0.1
Permanent AF	15 (31.9)	7 (36.8)	8 (28.6)	0.6

The values are presented as number (%), median (IQR).

SND, sick node disease; AVB, atrioventricular block; PM, pacemaker; ICD, implantable cardioverter-defibrillator; CRT-P, cardiac resynchronization therapy pacemaker; CRT-D, cardiac resynchronization therapy defibrillator; LVEF, left ventricular ejection fraction; CHF, chronic heart failure; CCD, chronic coronary disease; AF, atrial fibrillation.

### Lead removal results

Forty-six (97.9%) leads were lumenless leads (LLLs; Select Secure 3830, Medtronic Inc, Minneapolis, MN), and one stylet-driven lead (SDL; Solia S, Biotornik, SE&Co., KG, Germany). In 37 procedures, only the CSP lead was removed, and in 10, all implanted leads were removed. Forty (85.1%) leads were attempted to remove from the HB and seven from the LBB area. The median dwell time of the leads was 13.00 (5.00–40.75) months. In Group 1, the median dwell time was 4.00 (1.25–6.00) months, and in Group 2, it was 29.00 (14.50–48.00) months. Fifteen extracted leads were older than 24 months, and the most aged extracted lead was implanted for 7 years. The median dwell time of HBP leads was 13.50 (6.00–43.50) months and 2.00 (1.00–14.75) months for LBBAP leads. Three LBBAP leads were extracted after 1 year.

**Figure 1:**
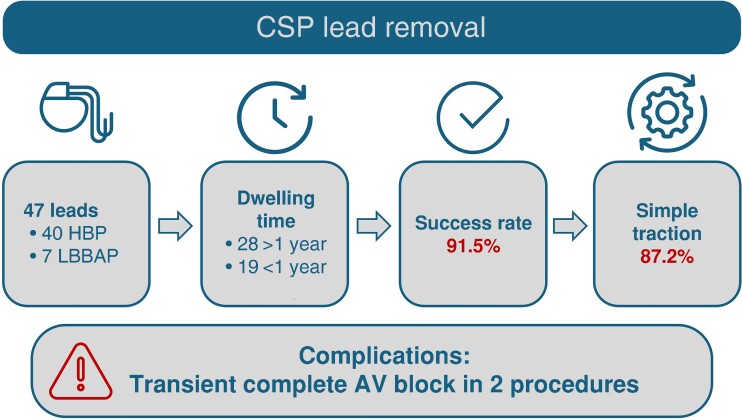
Central illustration. CSP, conduction system pacing; HBP, His bundle pacing; LBBAP, left bundle branch area pacing; AV, atrioventricular.

**Figure 2:**
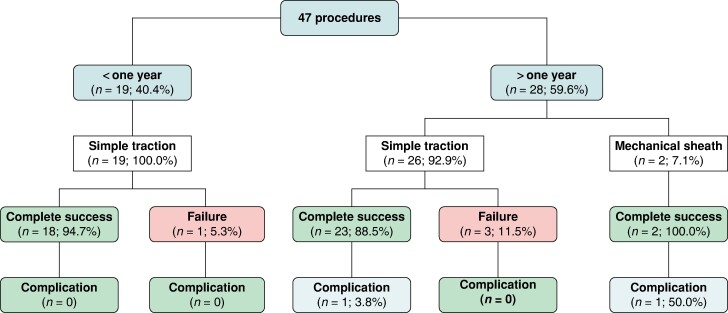
Procedural outcomes of conduction system pacing lead extractions.

**Figure 3:**
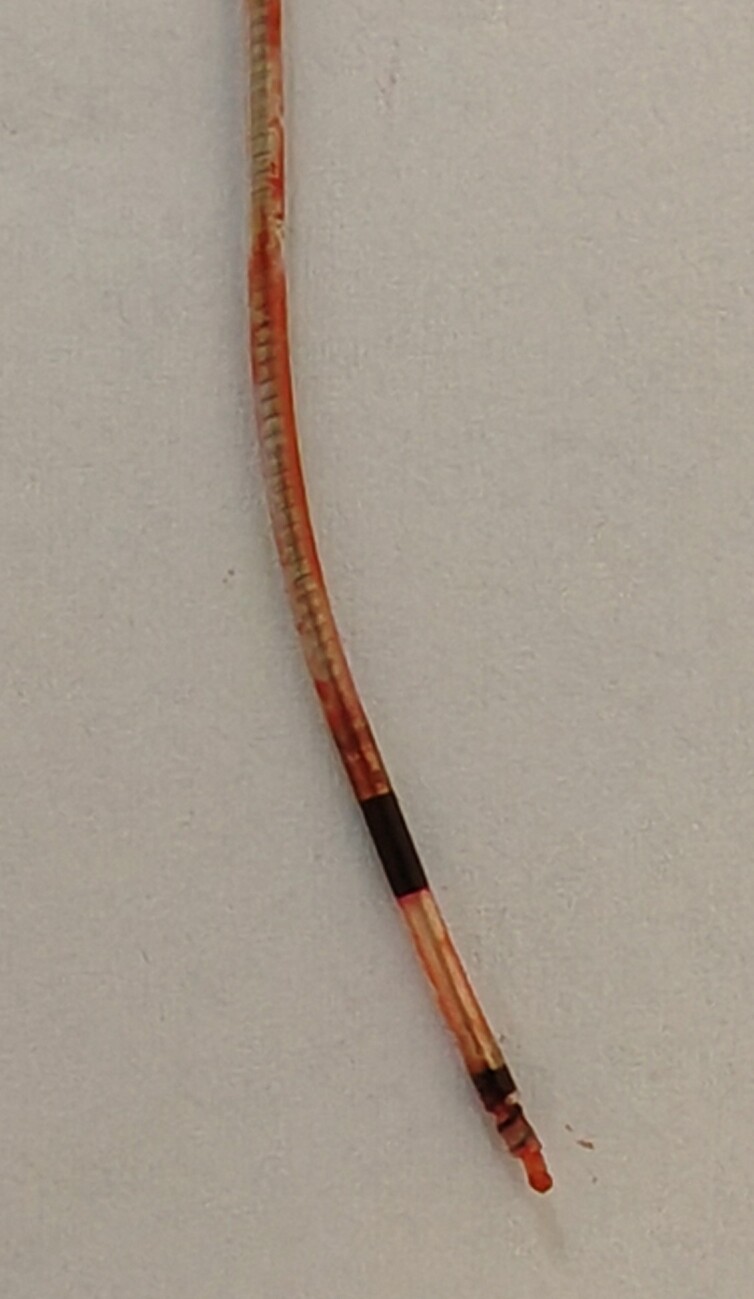
Extracted lead.

Overall, 43 (91.5%) leads were successfully removed, and 41 (87.2%) were removed with traction only (*Figure 1*). The procedural outcomes are presented in *Figure 2*. In two patients, after 24 and 55 months, the HBP leads were extracted with simple traction, and it was necessary to use mechanical sheaths to extract the right atrial and right ventricle (RV) leads. In one patient with a lead fracture in the subclavian region (32 months after implant), one fragment of the HBP lead was removed from the subclavian vein with traction only, and the intracardiac fragment was removed with a side retrieval snare loop (Needle’s Eye Snare, Cook Medical, Leechburg, PA, USA). All the leads were removed without any damage to the lead body or active fixation screw, with only minor tissue attachment (*Figure 3*).

Twelve (25.5%) extraction procedures were performed during device replacement due to battery depletion after 48.00 (43.50–60.50) months. Simple traction was used in 11 procedures. Complete procedural success was achieved in 75.0%, and in all 12 patients, the new CSP lead was reimplanted, 4 (33.3%) into HB and 8 (66.7%) into the LBB area.

All the unsuccessful procedures involved the HBP lead. In two patients, the leads were removed from the HB region but were trapped in the subclavian vein. In two remaining unsuccessful procedures, 2 and 48 months from implantation, extensive fibrosis in the brachiocephalic vein was the cause of failure. In two cases, we abandoned leads due to a high risk for lead extraction or lack of patient consent for lead extraction.

During two procedures (4.3%), one with simple traction and one with mechanical sheath, a complete atrioventricular (AV) block occurred. One patient had LBB block at baseline, and the other had normal AV conduction. In both cases, it was resolved during the procedure. No permanent injury to His–Purkinje was observed, and no other complications were noted.

In 27 out of 31 attempts (87.1%), new CSP leads were implanted: nine (33.6%) HBP leads and 18 (66.7%) LBBAP leads. In four cases, CSP lead implantation was attempted but was unsuccessful. In 11 (23.4%) patients, RV lead was implanted, and in seven (14.9%), left ventricle lead. After reassessing the indications, the pacemaker was not implanted in two cases (4.3%); in both cases the lead was removed due to infection.

## Discussion

This study presents data on the removal of CSP leads. Our results show that lead extraction from the His–Purkinje conduction system is safe and successful.

The data on CSP lead removal in adult subjects are sparse. Lead extraction from the His–Purkinje conduction system can potentially be related to specific complications, like injury to the conduction system, AV or ventricular septum perforation, and specific technical challenges with lumenless leads. The design of 3830 lead impedes the use of locking stylets during the lead removal procedure. However, other distinct features of the 3830 lead, including small diameter and related to its small tip surface area, and high tensile strength resulting from the inner cable extending to the tip, can facilitate lead extraction and better transfer of rotational forces. The high tensile strength of the 3830 lead also allows the use of powered tools, like lead extenders, without the need for a locking stylet. It was reported that lead design may affect the outcomes of lead extraction procedures.^11^

In our study population, all the removed leads were Medtronic Select Secure 3830 lumenless leads except for one. The success rate of complete lead removal was 91.5%. Most of the leads were extracted with simple traction (87.2%). Similar results were presented by Vijayaraman,^12^ with 95% success in lead extraction and the need to use extraction tools in 13% of patients. A high success rate of lead extraction was also reported by Boczar *et al.*^13^ in a small group; however, they had to use mechanical extraction tools in 44% of procedures.

The high tensile strength and non-retractable helix of the Medtronic 3830 lead have raised concerns about the possible myocardial avulsion and safety of lead removal.^14^ In our study cohort, transient AV block occurred during two procedures. A transient AV block can potentially be expected during lead extraction from the conduction system, and it can be regarded as a minor complication because of no persistent disability.^15^ Transient nature, quick resolution, and no new permanent conduction abnormalities suggest that the observed transient AV block resulted from mechanical stress on the HB or right bundle branch and did not result from direct injury to the His–Purkinje conduction system. A higher incidence of AV block in our study than during extraction of standard leads^16^ makes it reasonable to apply temporary pacing, especially in patients with baseline LBB block or when a mechanical sheath is used, and therefore forces implied on conduction tissue are more remarkable. No major complications occurred in our study. No complications were reported in studies by Vijayaraman and Boczar. No new atrioventricular conduction abnormalities explain the high success of reimplantation of CSP leads.

In our study, the main indication for lead removal was the high pacing threshold. In 25.5% of cases, the lead was extracted during the device replacement procedure. Thus, our high percentage of successful lead extraction and CSP reimplantation performed as an LBBAP made HBP lead extraction during device replacement a valuable option for patients with high pacing thresholds.

Data on CSP lead extraction were limited when LBBAP was initially performed.^17,18^ In our study, seven leads were removed from the LBB area, and the oldest extracted lead dwell time was 26 months. All the LBBAP leads were removed using traction only and without any complication. Similar results were reported in a case report studies.^17–19^ However, due to the small number and short dwelling time of removed LBBAP leads, our results of LBBAP lead removal need to be corroborated in a larger series.

The success rate of lead extraction by traction only for conventional pacing leads was reported to be 27.3–45.1%.^3,15^ In our study, it was successful in 87% of extracted leads, but due to the small cohort, it was impossible to determine if the results were only the consequence of a shorter lead dwelling time or if they represented the perpendicular course of the CSP lead in relation to the septum, which reduced fibrotic lead encapsulation. The main reason for the failure of the lead extraction was lead entrapment in the subclavian vein. Despite the high effectiveness of simple traction in HBP lead extractions, the use of passive sheath as a primary approach should be considered in patients with severely compromised subclavian vein patency.

Small populations analysed in our study and in previously published studies as well as short lead dwelling time make it difficult to draw definitive conclusions and directly compare these results with outcomes of standard pacing lead extraction.^3,20^ Yet, our results show that LLC lead extraction from the conduction system can be safe and potentially less challenging than standard pacing lead extractions. Further empirical evidence derived from studies encompassing larger study populations with longer lead dwelling times is required to substantiate the results of our research.

## Study limitations

Our study represented a single-centre, observational analysis. Only one SDL lead was removed in our study, and the results of LLC lead extraction from the conduction system could not be directly translated to SDL lead removal. Furthermore, HBP for CSP is losing importance to LBBAP, and most of the removed leads in our study were HBP leads. For many years, however, HBP had been the only technique for CSP, and the need for HBP lead extraction might rise in the near future.

## Data Availability

The data underlying this article will be shared on reasonable request to the corresponding author.
